# Characterization of Spatial Frequency Channels Underlying Disparity Sensitivity by Factor Analysis of Population Data

**DOI:** 10.3389/fncom.2017.00063

**Published:** 2017-07-11

**Authors:** Alexandre Reynaud, Robert F. Hess

**Affiliations:** McGill Vision Research, Department of Ophthalmology, McGill University Montreal, QC, Canada

**Keywords:** disparity sensitivity, qDSF, binocular vision, stereopsis, individual differences, factor analysis

## Abstract

It has been suggested that at least two mechanisms mediate disparity processing, one for coarse and one for fine disparities. Here we analyze individual differences in our previously measured normative dataset on the disparity sensitivity as a function of spatial frequency of 61 observers to assess the tuning of the spatial frequency channels underlying disparity sensitivity for oblique corrugations (Reynaud et al., 2015). Inter-correlations and factor analysis of the population data revealed two spatial frequency channels for disparity sensitivity: one tuned to high spatial frequencies and one tuned to low spatial frequencies. Our results confirm that disparity is encoded by spatial frequency channels of different sensitivities tuned to different ranges of corrugation frequencies.

## Introduction

The visual system utilizes the displacement or disparity in the two images seen by the two eyes to compute the depth of objects. In terms of the underlying mechanisms, Pulliam (1982) first suggested that there were two global disparity mechanisms, one tuned to low spatial frequencies involving coarse disparities and one tuned to high spatial frequencies involving fine disparities. Yang and Blake (1991) also argued for only two spatial frequency channels for disparity processing and their model was later refined by Tyler et al. (1994). Additional evidence for two spatial frequency channels subserving disparity processing comes from the work of Norcia et al. (1985); Wilcox and Allison (2009); Witz et al. (2014). However, other studies suggest a multiple channels model (Julesz and Miller, 1975; Glennerster and Parker, 1997; Serrano-Pedraza et al., 2013).

Assessing the tuning of these channels has been of great importance for mechanistic models of stereo computer vision (Marr and Poggio, 1979; Nishihara, 1984; Quam, 1987; Rohaly and Wilson, 1993). These can be used to map different scales of matching in hierarchical structures (Nishihara, 1984; Quam, 1987) with, for instance, coarse-to-fine constraints (Rohaly and Wilson, 1993). In robotic vision, these tuning properties can be used to calibrate cameras (Tsai, 1986) and vergence algorithms (Piater et al., 1999; Lonini et al., 2013).

**Figure 1:**
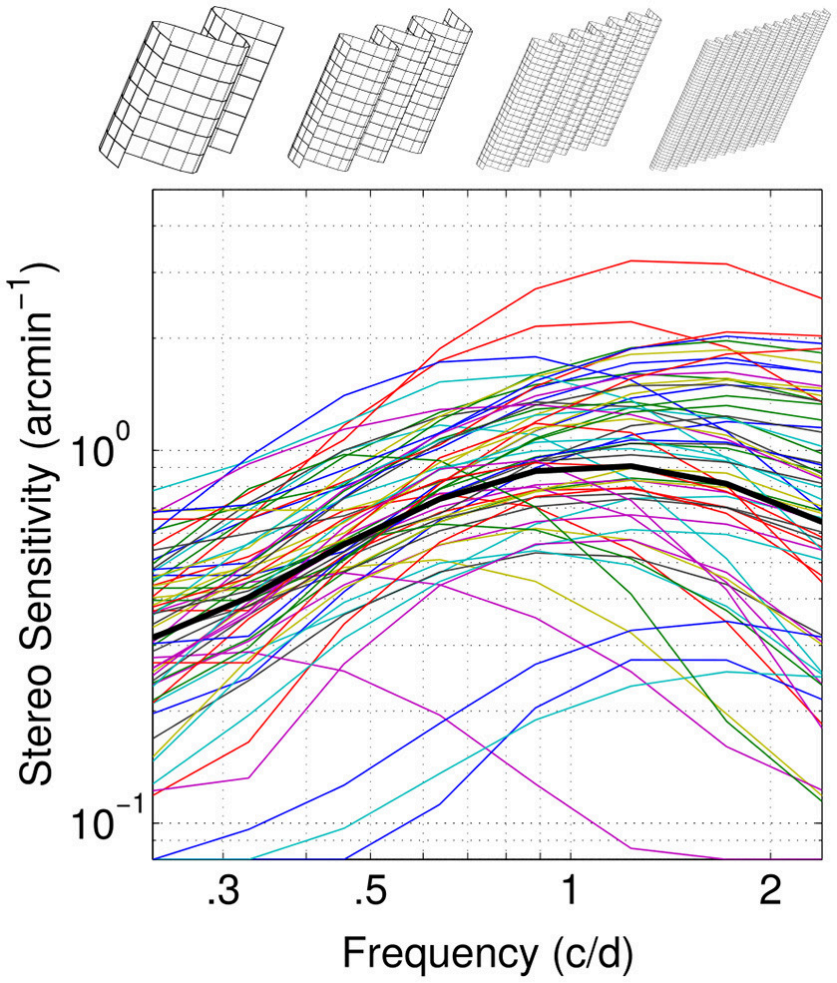
Normative dataset. Disparity sensitivity as a function of spatial frequency is reported for 61 individual observers (thin color lines) and their average (thick black line). Sketches at the top illustrate the stimulus at different corrugations frequencies. Adapted with permission from Reynaud et al. (2015).

While most studies have used masking paradigms to characterize spatial frequency channels for stereopsis (Julesz and Miller, 1975; Yang and Blake, 1991; Shioiri et al., 1994; Tyler et al., 1994; Glennerster and Parker, 1997; Prince et al., 1998; Serrano-Pedraza et al., 2013), another possibility comes from factor analysis of population data (Read et al., 2016). The individual differences are then treated as systematic and meaningful, reflecting the true variability of underlying mechanisms rather than random noise (Peterzell, 2016). Identifying the sources of variability within the population will inform on the common processing mechanisms. Therefore, spatial and temporal frequency channels can be characterized by analyzing individual differences and correlations. The rationale is that the correlation in detection thresholds for pairs of stimuli should be higher for stimuli detected by the same mechanism than for stimuli detected by different mechanisms (Owsley et al., 1983; Sekuler et al., 1984; Billock and Harding, 1996). Hence by looking at the inter-correlations between individuals' sensitivity at neighboring frequencies, one is able to determine the presence of frequency channels (Mayer et al., 1995; Billock and Harding, 1996; Peterzell and Teller, 2000; Simpson and McFadden, 2005; Rosli et al., 2009). Therefore, a factor analysis of the dataset consisting of a principal component analysis (PCA) and a rotation of the factors in order to determine a simple structure can characterize the tuning curves of the channels (Simpson and McFadden, 2005). Using factor analytics within the population sensitivities Peterzell and Teller (1996, 2000) assessed spatial frequency channels tuning for luminance and color contrast sensitivities. Here we use similar methods to analyze individual differences in our previously measured normative dataset on disparity sensitivity as a function of spatial frequency for oblique corrugations of 61 observers (Figure 1; Reynaud et al., 2015) in order to assess the spatial frequency tuning of the underlying disparity channels.

## Methods

In this paper, we analyze the normative dataset for the disparity sensitivity as a function of spatial frequency of 61 observers (25 males, 36 females, mean age 26 years, ±5.7 SD, with normal or corrected to normal-visual acuity) we measured previously using the quick Disparity Sensitivity Function (*qDSF*, Reynaud et al., 2015), a method adapted from the quick Contrast Sensitivity Function (*qCSF*, Lesmes et al., 2010).

The stimuli used in this dataset were stereograms composed of spatially filtered 2-D fractal noise carriers with oblique (45° or 135°) sinusoidal corrugations at 0.24, 0.33, 0.46, 0.64, 0.89, 1.23, 1.72, and 2.39 c/d. The spatial frequency of the carrier was 4 times the spatial frequency of the corrugation (see Reynaud et al., 2015). Disparity was modulated and the subjects' task was to identify the orientation of the corrugation in depth (45° or 135°) in a single-interval identification task to measure the disparity detection threshold. Stimuli were displayed on a passive wide 23″ 3D-Ready LED monitor ViewSonic V3D231, viewed with polarized 3D glasses at 70 cm, in a dim-lit room. Measured individual disparity sensitivity functions as a function of spatial frequency and their average are reproduced in Figure 1. Analysis was performed with Matlab R2016a (The MathWorks). The hierarchical clustering analysis was specifically performed with the statistics and machine learning toolboxes functions.

## Results

The average disparity sensitivity peaks are in the high spatial frequency range, around 1.2 c/d. However, we can observe a large variability in the individual sensitivities: some showing a low-pass, band-pass or high-pass profiles (Figure 1). Hence a factor analysis of these sensitivities might provide insight into the common mechanisms mediating them.

**Figure 2:**
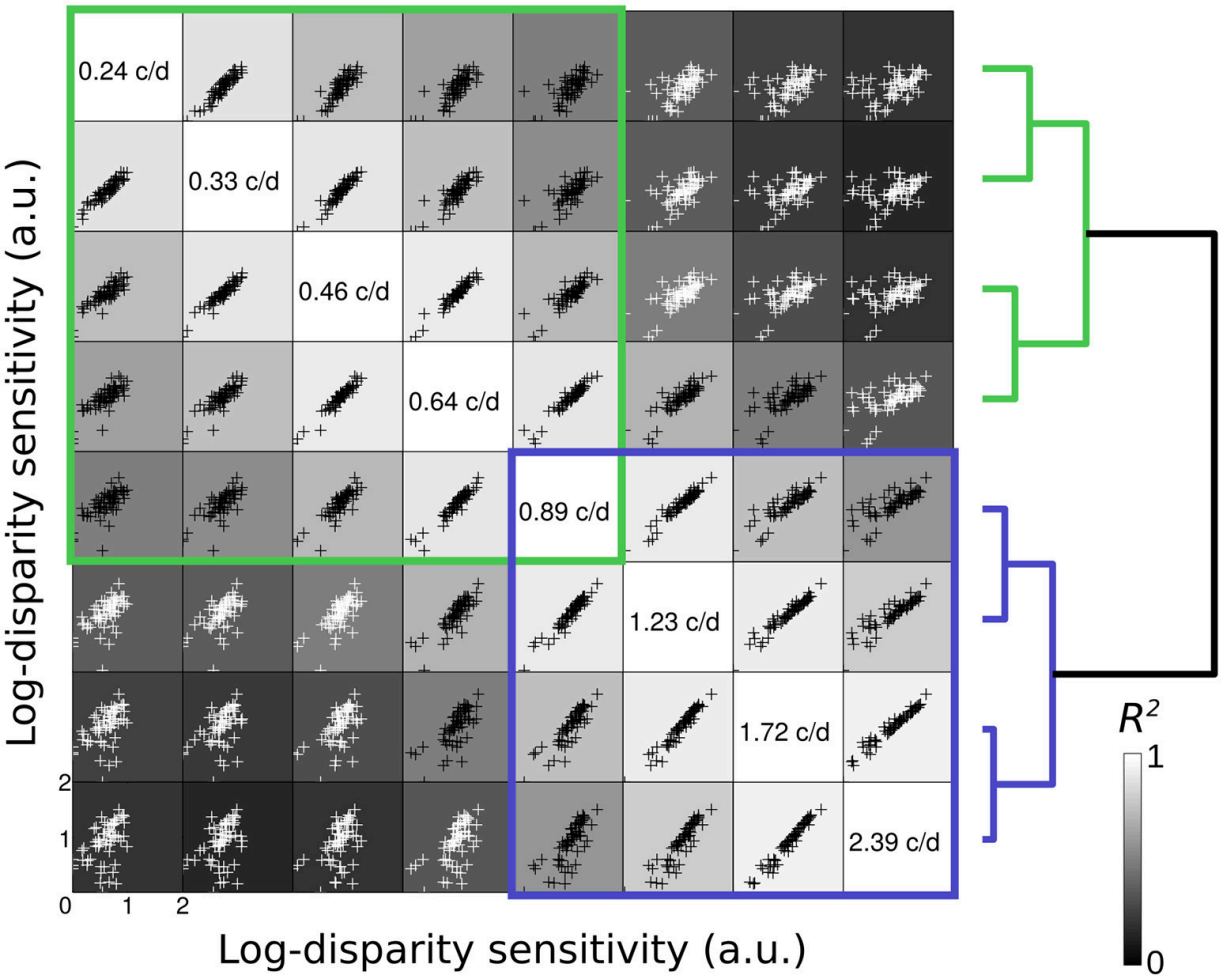
Scatterplot matrix of inter-correlations. In each cell, the scatterplot represent the inter-correlation of the log-disparity sensitivity (arbitrary units) of all 61 observer at one frequency (frequency indicated on the diagonal in the same row) as a function of their sensitivity at another frequency (frequency indicated on the diagonal in the same column). The shade of the background in each cell indicates the value of the coefficient of determination *R*^2^ between the two frequencies (from black = 0 to white = 1). Black datapoints indicate *R*^2^ > 0.5 and white datapoints *R*^2^ < 0.5. Blue and green squares highlight regions of high inter-correlations. On the right is represented the classification dendrogram of the spatial frequencies. The pairwise distance was calculated as one minus the sample linear correlation between observations and the hierarchical cluster tree was computed with the average distance.

Figure 2 represents the scatterplot matrix of inter-correlations (Peterzell, 2016) for log-disparity sensitivity of all 61 observers. In each cell within the figure, the scatterplot represent the inter-correlation of the log-disparity sensitivity of all observers at one frequency (frequency indicated on the diagonal in the same row) as a function of their sensitivity at another frequency (frequency indicated on the diagonal in the same column) are depicted. For instance, in the bottom-left cell, the log-disparity sensitivity of each observer at 0.24 c/d is plotted pairwise against its log-disparity sensitivity at 2.39 c/d. Then the coefficient of determination *R*^2^ between the two frequencies is computed. Two regions of high inter-correlations (*R*^2^ > 0.5) at low spatial frequency (green) and high spatial frequency (blue) appear along the diagonal.

These two regions are supported by the hierarchical clustering analysis of the log-disparity sensitivity at all spatial frequencies. The pairwise distance between observations was calculated as one minus the sample linear correlation between observations and the hierarchical cluster tree was computed with the average distance. The resulting dendrogram is represented at the right of the inter-correlation matrix, with each spatial frequency being the leaves. Nevertheless, we can note that different distance measures and different linkage procedures can result in relatively different final clusters, some grouping the 3 lowest and 5 highest frequencies for instance. The two cluster branches whose linkage is less than the default 70% are represented in blue and green. As for the first qualitative approach, these two groups suggest the presence of two spatial frequency channels for disparity sensitivity, which might correspond to the coarse and fine disparity channels.

**Figure 3:**
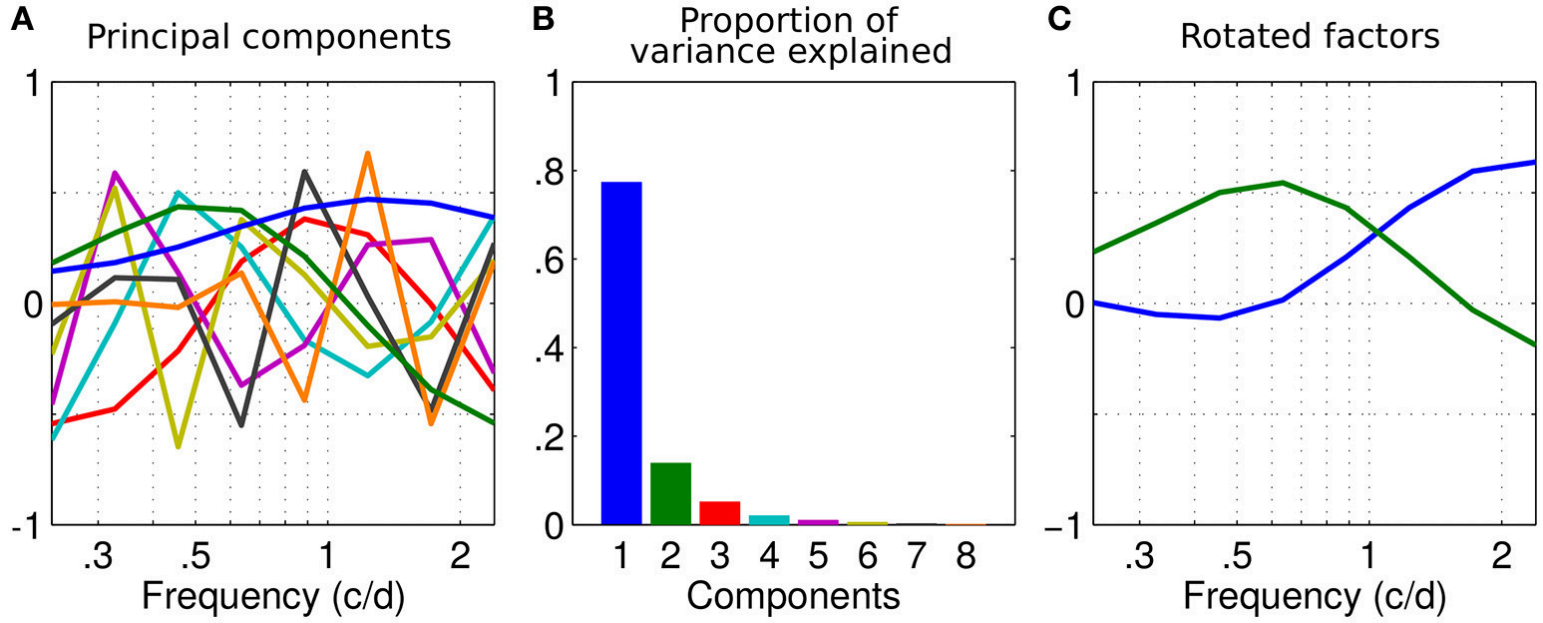
Factor analysis. **(A)** Principal components of the dataset as a function of spatial frequency. Their order is indicated by colors in **(B)**. **(B)** Scree plot of the variance explained by each component of the principal component analysis (PCA) in **(A)**. **(C)** First two components rotated using a varimax rotation.

In order to determine the precise tuning of these channels, we performed a factor analysis on the dataset. If we decompose the full dataset with a principal component analysis (PCA), we obtain the components shown in Figure 3A, with a percentage of explained variance (calculated from the eigenvalues of the PCA) associated with each component reported in the scree plot Figure 3B.

The first component has the shape of the average sensitivity (see Figure 1). The two first components (blue and green) explain more than 91% of the variance and the elbow of the scree plot occurs between the second and third components (Figure 3B). As we previously identified two regions of high inter-correlations and that this percentage of explained variance is considered enough to accurately describe the data (Simpson and McFadden, 2005), these two principal components were picked to describe the underlying disparity sensitivity channels. In order to make sense of them, these two principal components, or factors, were then rotated using a varimax orthogonal rotation to obtain a simple structure accounting for the channel tuning curves (Kaiser, 1958; Peterzell and Teller, 2000; Simpson and McFadden, 2005; Peterzell, 2016). These factors-tuning curves are reported in Figure 3C. The first factor peaks at the highest measured frequency 2.4 c/d and the second peaks around 0.65 c/d. They characterize the high and low spatial frequency channels identified by the inter-correlation analysis (respectively blue and green regions in Figure 2).

We wanted to test if the two channels we identified could in fact account for different classes within the population. In order to estimate the weights β of each of these factors in each individual sensitivity, we projected our dataset onto the basis defined by the two identified factors. The best linear unbiased estimator of β is obtained using the Moore-Penrose pseudo inverse X^+^ (equation 1):

(1) $$\beta ={\text{X}}^{+}\text{y}$$

where y is the matrix of all individual sensitivities, X^+^ is the Moore-Penrose pseudo inverse of the new basis matrix X whose two columns represent the two factors and β is a two-rows matrix in wihich each column contains the pair of weights associated to the two factors estimated for each subject (Friston et al., 1995; Woolrich et al., 2004; Reynaud et al., 2011).

**Figure 4:**
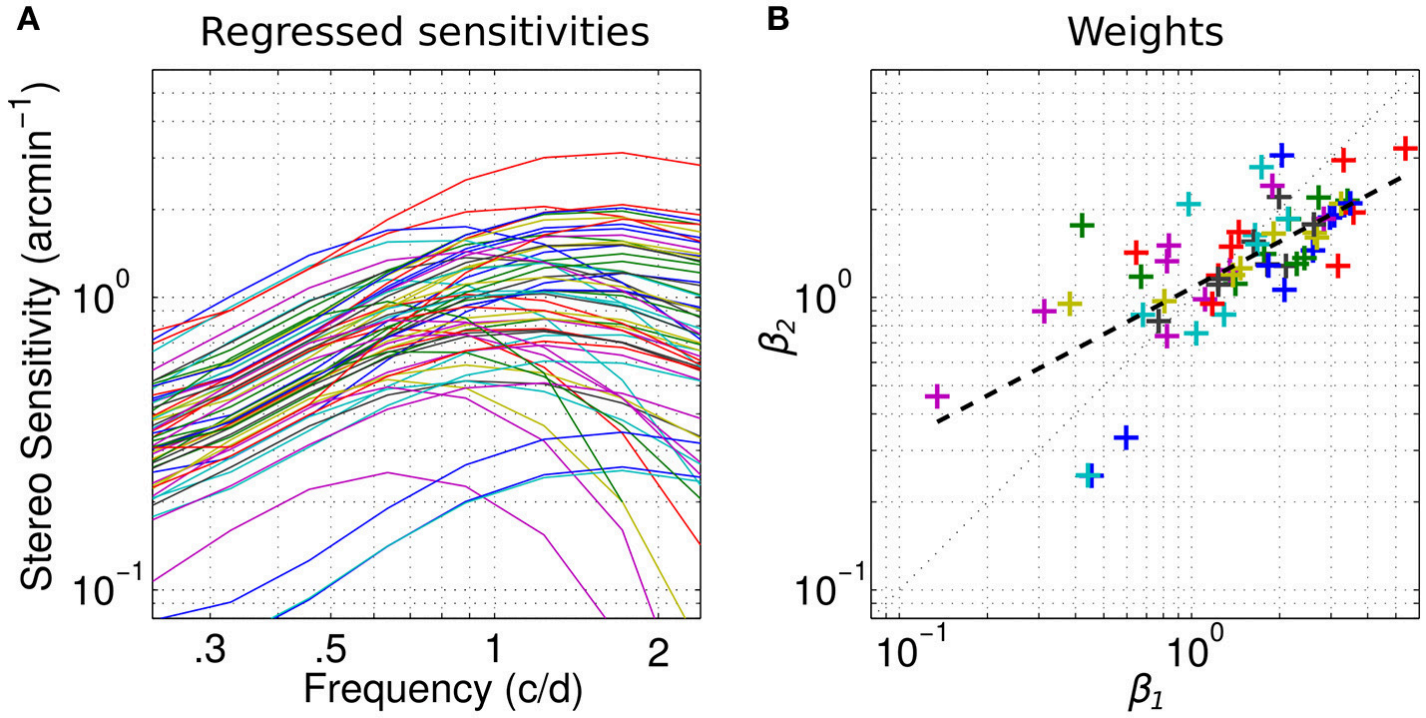
Channels weights. **(A)** Individual sensitivities replotted using only the two channels factors. Same color-code as in Figure 1. **(B)** Scatterplot of the weights of the first factor β_1_ vs. the weights of the second factor β_2_ for all observers. Dashed line indicates linear regression on the log-values of the weights.

The sensitivities ŷ reconstructed solely from the linear combination of these two factors are plotted in Figure 4A (Equation 2):

(2) $$\begin{array}{c} \hat{y}=\text{X}\beta \end{array}$$

We can see that they overall faithfully reproduce the original sensitivities except for the very low-pass profiles whose peaks shift to the right.

To determine whether these channels can account for different classes within the population, we report a scatterplot of the weights β_1_ of the first factor vs. the weights β_2_ of the second factor in Figure 4B for all observers. The mean weights for the first and second factor are, respectively, 1.76 and 1.48. As expected from the explained variance (Figure 3B), the weight of the first factor—the high-frequency channel—is greater than the weight of the second—the low frequency channel—in 70% of the cases. The distribution of these weights appears homogeneous and no clusters are revealed. However, the weights of the first factor seem to be relatively greater than the weights of the second in the high values range whereas it seems to be slightly the opposite in the low values range. This is further revealed by the slope of the linear regression between the log-values of the weights 0.53, which is inferior to 1 (dashed line). In fact, the correlation between the weight is very high (coefficient of determination *R*^2^ = 0.51, *p* < 0.0001). Altogether, these observations suggest that the weight of the low and high spatial frequency channels co-vary: when the sensitivity is high for the low frequency channel, it is high for the high frequency channel too. But the high frequency channel contributes relatively more when the sensitivity is high and the low-frequency channel contributes relatively more when the sensitivity is low, in accordance with our previous observations (Reynaud et al., 2015).

## Discussion

The qDSF method assumes the sensitivity function follows the truncated log-parabola model and hence has a bell shape with a constant part, an increase to a peak and a drop-off (Watson and Robson, 1981; Lesmes et al., 2010). We previously showed that this model can accurately represent the sensitivity function compared to non-constrained methods (Reynaud et al., 2015) and documents large differences in sensitivities within the population (see Figure 1). For different individuals, this function can peak at very different frequencies and can show low-pass, band-pass or high-pass profiles. The resultant variability in sensitivity across spatial frequency provides a rich dataset for inter-correlation analyses (Peterzell et al., 1995; Peterzell, 2016).

Because two regions of inter correlations were identified among the population in Figure 1 and because 2 components accounted for more than 91% of the variance, our data could accurately be described by just 2 channels. However, the criterion to select the number of meaningful components in a PCA may vary. Popular selection methods such as a scree plot (Jackson, 1993) or the Random average under permutation analysis will indeed determine 2 components while some other methods will give less (the broken stick method gives 1 component) or more (the parallel analysis gives barely 3, the kaiser Guttman criterion which recommends eigenvalues >1 gives 3 too). Some methods such as the Bartlett tests even recommends all the 8 components which would not reduce the dimensionality of the data (Bartlett, 1950). A complete description of these methods can be found in Peres-Neto et al. (2005).

Hence, we cannot completely rule out the possibility of a single-channel or multiple-channels hypothesis. Serrano-Pedraza and Read reported a single channel mechanism specific to vertical corrugations (Serrano-Pedraza and Read, 2010, though see Witz et al., 2014). However, the large difference we can observe between the lowpass profile of sensitivity for some observers compared to the bandpass of other ones would indicate that more than one channel are involved. Several studies suggested a multiple-channels mechanism (Julesz and Miller, 1975; Schumer and Ganz, 1979; Cobo-Lewis and Yeh, 1994; Glennerster and Parker, 1997; Serrano-Pedraza et al., 2013) with a broad channel tuning of ~2–3 octaves, comparable to our observations (Schumer and Ganz, 1979; Cobo-Lewis and Yeh, 1994). It is then possible that the 2 channels we observe are part of a multiple-channels system covering a wider range of spatial frequencies or could also overlap with intermediate channels continuously covering the spatial frequency range. Yang and Blake (1991) also observed two spatial frequency channels for disparity sensitivity using a masking paradigm. They described one channel centered around 3 c/d which could correspond to the high spatial frequency channel we observed and one centered around 5 c/d. However, their study and the present study didn't measure the same spatial frequency range which might explain why they didn't identify our low spatial frequency channel and why we didn't observe their high one.

The results of the present study suggests that there are two channels (Figure 4B), a low frequency channel that contributes to the detection of low corrugation frequencies and a more sensitive high frequency channel that contributes to the detection of high corrugation frequencies. We didn't observe any dichotomy based on these two channels within our population (Wilcox and Allison, 2009) which confirms the observations of most other population studies (Coutant and Westheimer, 1993; Bohr and Read, 2013; Bosten et al., 2015).

The implications of the assessment of the tuning of these disparity channels could be important in computer vision to design behaviorally relevant stereo matching algorithms. For instance, it could be used to tune the different layers of multi-scale algorithms (Rohaly and Wilson, 1993) or provide fine and coarse scales for algorithms processing in center and periphery, respectively, as stereopsis could be mediated by different mechanisms in central and peripheral vision (Wardle et al., 2012; Witz and Hess, 2013).

## Conclusion

The analysis of the inter-correlations in the disparity sensitivity as a function of the spatial frequency, revealed two disparity channels. With a factor analysis of the population data, we determined that the first channel is tuned to high spatial frequencies (peaks at 2.4 c/d) and the second is tuned to low spatial frequencies (peaks at 0.65 c/d). We also observed that these two channels are well correlated with each other. Our results confirm that disparity is encoded by multiple spatial frequency channels that are of different sensitivities and subserve different ranges of corrugation frequencies.

## Author contributions

AR and RH designed the research and wrote the manuscript. AR analyzed the data.

### Conflict of interest statement

The authors declare that the research was conducted in the absence of any commercial or financial relationships that could be construed as a potential conflict of interest.
